# Intraguild Predation and Chemical Cue Responses Between *Phytoseiulus persimilis* and *Neoseiulus californicus* in Laboratory Assays

**DOI:** 10.3390/insects17020157

**Published:** 2026-01-31

**Authors:** Fatma Sh. Kalmosh, Bo Zhang, Nikola Đukić, Abdulaziz Alamri, Salman Alrokayan, Xuenong Xu

**Affiliations:** 1 Department of Field Crops and Cotton Mites, Plant Protection Research Institute, Agricultural Research Center, Dokki, Giza 12618, Egypt; 2 State Key Laboratory for Biology of Plant Diseases and Insect Pests, Institute of Plant Protection, Chinese Academy of Agricultural Sciences, Beijing 100193, China; zhangbo05@caas.cn; 3 Institute for Medicinal Plants Research “Dr. Josif Pančić”, 11000 Belgrade, Serbia; nikoladjukadj@yahoo.com; 4 Biochemistry Department, College of Science, King Saud University, P.O. Box 2455, Riyadh 11451, Saudi Arabia; abalamri@ksu.edu.sa; 5 Research Chair of Biomedical Applications of Nanomaterials, Biochemistry Department, College of Science, King Saud University, P.O. Box 2455, Riyadh 11451, Saudi Arabia; salrokayan@ksu.edu.sa

**Keywords:** intraguild predation, predatory mites, foraging behavior, chemical cues, predator–prey–plant interactions

## Abstract

Species sharing the same trophic level can interact not only through competition for resources but also through intraguild predation (IGP). Therefore, an important step toward implementing successful multiple predator releases in biological control strategies requires resolving how predators respond to the presence of heterogeneous competitors. This study examined the compatibility of two predatory mites, *N. californicus* and *P. persimilis*, which are both widely employed to suppress two-spotted spider mite populations in greenhouses and open fields. The experiments quantified the frequency and intensity of IGP across different developmental stages of these species on bean leaves, considering scenarios both with and without their shared prey being present. Additionally, a Y-tube olfactometer was employed to assess whether either predator avoided prey patches previously occupied by other heterospecifics, thereby providing insights into potential chemical cues that influence predator behavior. The results revealed that adult females of both predatory mite species predominantly targeted heterospecific eggs and larvae, whereas adults were largely avoided. In the absence of shared prey, *N. californicus* attacked 83% of the *P. persimilis* larvae and 37% of the eggs, whereas *P. persimilis* consumed 67% of the *N. californicus* eggs. The presence of shared prey reduced IGP risk by approximately 60%. Olfactometer assays revealed no significant avoidance of plants inhabited by heterospecifics at densities of 20 or 40 adults; both predators were similarly attracted to herbivore-induced volatiles. Generalized linear models indicated that host plant experience had a significant influence on the foraging response of *N. californicus*, whereas the effects of the feeding state weakened over time. Understanding predator foraging plasticity and responsiveness to chemical cues can help optimize biological control strategies in complex agroecosystems.

## Simple Summary

Farmers are increasingly relying on natural enemies instead of pesticides for crop protection; however, using multiple predator species simultaneously can lead to unexpected interactions that may reduce the effectiveness of pest control. This study investigated the interaction between two commonly used predatory mites when they share the same food source, the two-spotted spider mite, and how plant volatiles influence their behavior. The aim was to determine whether these predators attack each other, avoid one another, or alter their foraging behavior when they occur together. Laboratory experiments showed that both predatory mite species primarily attacked the eggs and larval stages of the other species in the absence of shared prey, but such intraguild attacks were greatly reduced when spider mite prey were available. Both predators were attracted to herbivore-damaged plants and typically did not avoid plants occupied by the other predator. Previous experience had short-term effects on their behavior. Overall, the findings suggest that these two predators can be safely used together when enough prey densities are present. This knowledge contributes to the development of sustainable pest management strategies, reduces pesticide use, and supports environmentally friendly agriculture.

## 1. Introduction

Owing to the widespread success of biological control in many crops, particularly in greenhouses [1,2], the use of multiple natural enemies against different pests has transformed crop systems from relatively simple predator–pest–plant interactions into complex food-web structures [3,4]. As these artificial food webs become more complex, indirect interactions become increasingly important in shaping pest dynamics and regulating natural enemy populations [3]. When different predatory mite species are drawn to plants infested with the same prey, their interactions may involve food competition, intraguild predation, or behavioral interference, all of which can influence foraging behavior [5,6,7]. Intraguild predation is particularly likely in ecosystems that use generalist predators, as they consume not only the pest but also may prey on other natural enemies present in the same habitat [2,5,8]. Predators can avoid each other’s presence, resulting in their temporal or spatial separation, to avoid such antagonistic consequences [9]. This may reduce their ability to control herbivore populations.

Predatory mites (Acari: Phytoseiidae) constitute a vast number of species that are widely utilized as biological control agents in numerous agricultural systems worldwide, particularly for controlling species of phytophagous mites and small insects [10]. Many commercial companies produce these species. *Phytoseiulus persimilis* Athias-Henriot (Acari, Phytoseiidae) is one of the most common biocontrol agents; it is a specialized mite predator that is mass-produced and marketed for the control of Tetranychidae mites (e.g., two-spotted spider mite, *Tetranychus urticae* Koch), (Acari: Tetranychidae), which are among the most damaging pests, attacking over 1100 plant species and capable of rapidly developing resistance to many pesticides [11,12,13]. *Neoseiulus californicus* (McGregor) (Acari: Phytoseiidae) is a selective predator of Tetranychid mite species [14]. Moreover, *N. californicus* can also feed and reproduce on plant-derived kinds of food sources, such as small pest species (thrips, mites, and whiteflies), as well as various types of pollen [15,16]. This species also has a relatively high survival rate when the prey population density decreases [17] and displays notable resistance to starvation [18].

Chemical signals play a central role in the foraging behavior of predatory mites [19,20]. However, both *P. persimilis* and *N. californicus* are attracted to volatiles emitted from spider mites [21]. Furthermore, these predators can potentially engage in interspecific predation [22,23]. In such interactions, one predator may induce antipredator behavior in heterospecific competitors, such as avoiding its presence and altering egg distribution and oviposition in response to signals from other species [7], thereby influencing the performance of predators in biological control programs [24,25]. Understanding of which prey species and prey type are favored by predators under specific situations, as well as how previous experiences with prey stimuli influence foraging behavior, remains limited. Recent research has suggested that maternal dietary experiences can significantly impact the foraging behavior of predatory mites later in life. Specifically, studies have shown that both maternal diet [26] and early-life dietary experiences [27,28,29] play critical roles in shaping these behaviors. Learning is a widespread phenomenon utilized by foraging predators to increase their ability to search for, recognize, and capture prey [30]. The behavioral changes resulting from prey experience are often specific to certain life stages and phases, with many animals exhibiting heightened sensitivity to environmental stimuli [28,31,32,33], particularly during their early life stages. This sensitivity is particularly notable in plant-inhabiting predatory mites such as *P. persimilis*, *N. californicus*, and *Amblyseius swirskii* [34,35], where early exposure to foraging cues can lead to significant and lasting changes in behavior. Research has shown that these mites, when exposed to prey cues during their early development, demonstrate improved prey-searching efficiency, enhanced recognition capabilities, and increased capture success compared with those that lack such cues [28,34,36,37]. This underscores the critical role of early life experiences in shaping foraging behavior, which can have important implications for the effectiveness of these predators as biological control agents in agricultural settings. Although intraguild predation (IGP) and volatile-mediated foraging have been studied in *P. persimilis* and *N. californicus*, existing research has typically examined these processes separately, under different experimental conditions, or without considering the role of early learning. As a result, important questions remain regarding how these two predators interact when sharing the same habitat and prey resources, and how chemical cues influence their foraging decisions.

To address these gaps, the present study aimed to investigate intraguild predation between different life stages of *N. californicus* and *P. persimilis* on bean leaves, with a focus on interactions in both the presence and absence of their shared prey, *T. urticae*. We also examined the innate and learned responses of these two predatory mite species. Specifically, we tested whether *P. persimilis* and *N. californicus* could distinguish between plants associated solely with prey and those associated with both prey and heterospecific predators. Additionally, we examined how early exposure to chemical cues from heterospecifics—representing early learning—impacts adult foraging behavior using a Y-tube olfactometer.

## 2. Materials and Methods

### 2.1. Test Mites

Lima bean (*Phaseolus vulgaris*) or cucumber (*Cucumis sativa*) plants were chosen as the host plants for rearing *T. urticae* in a climate-controlled environment set to 25 °C with a 16 h light cycle in the Laboratory of Predatory Mites, Institute of Plant Protection, Chinese Academy of Agricultural Sciences, Beijing. The seedlings were cultivated under standard cultivation practices until they developed two true leaves. Healthy plants were grown under these same conditions for three weeks before being introduced into the spider mite colonies every two weeks. Predatory mites, *P. persimilis* and *N. californicus*, were raised at 25 °C on detached cucumber or bean leaves infested with two-spotted spider mites, and were reared for multiple generations using *T. urticae* as their prey, for more than 10 years in the Laboratory of Predatory Mites, Institute of Plant Protection, Chinese Academy of Agricultural Sciences, Beijing, China, ensuring contamination-free cultures for five generations. These leaves were placed upside down on flowerpots inside water-filled trays and covered with Plexiglas containers to maintain humidity. Additionally, two to three leaves from the spider mite cultures were added every two weeks to support the predatory mite populations. The *P. persimilis* and *N. californicus* individuals originated from laboratory stock cultures maintained under similar conditions.

### 2.2. Intraguild Predation

To assess the rates of intraguild predation, adult female predatory mites were tested for their ability to feed on eggs, larvae, or adult females of a heterospecific species, both in the presence and absence of their shared prey, *T. urticae*. Bean leaf discs measuring 2.5 cm in diameter were positioned on water-saturated cotton wool inside plastic containers (8 cm tall, 7 cm in diameter at the top, and 4.5 cm at the base). On each clean leaf disc, one developmental stage of intraguild prey was introduced separately: five eggs, five larvae, or a single adult female from one species, which was mated and 3–5 days old, sourced from the laboratory culture. An adult female from the other species was subsequently added to the disc. After 24 h, the numbers of surviving and preyed-upon individuals were recorded. Additionally, we noted the number of mites that attempted to escape and were trapped in the moist cotton wool surrounding the leaf disc.

To assess intraguild predation in the presence of the shared prey, a fresh bean leaf was placed in a larger container (10 cm height, 14 cm top diameter, and 11.5 cm base diameter) and inoculated with 100 adults of *T. urticae*. After two days, 20 individuals of one developmental stage—either eggs or larvae—of one predatory mite species were added, ensuring that only a single stage was present during each trial. Simultaneously, 20 adult females of the second predatory mite species were introduced. Following 24 h, the numbers of surviving, killed, and escaped mites were recorded; each experiment was repeated three times with forty replicates. All the experiments were performed in a controlled climatic chamber maintained at 25 ± 2 °C and 60 ± 5% relative humidity.

### 2.3. Behavioral Bioassay

A Y-tube olfactometer (Institute of Plant Protection, Chinese Academy of Agricultural Science, Beijing, China), following the design described by [38], was employed to assess the behavioral responses of female predatory mites to volatiles released by heterospecific individuals. The device consisted of a glass Y-tube (27 cm in length, 3.5 cm in diameter) fitted with a central metal wire to serve as a guiding track for mite movement. The airflow was regulated and balanced through a flow meter equipped with needle valves positioned between the odor source chambers and the olfactometer arms.

**Figure 1:**
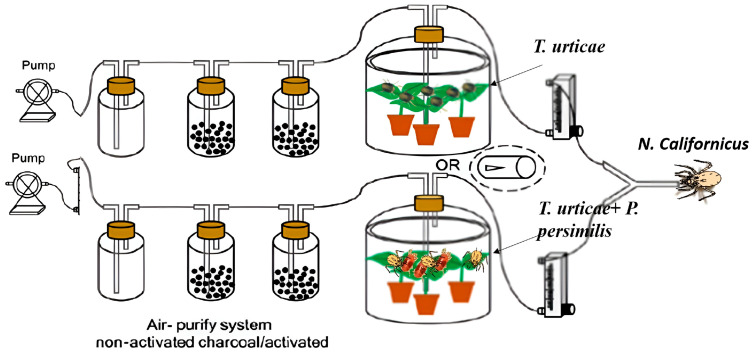
Schematic diagram of the predatory mite olfactory assays.

To ensure standardized conditions, the airflow in both arms was adjusted to 1.5 m s^−1^, which created two separate and stable odor fields at the branching point of the Y-tube. The boundary between these odor plumes coincided with the central guiding wire, ensuring clear orientation for the mites [38]. Distinct odor sources were alternately introduced into the two arms, enabling the predators to choose between them (Figure 1).

In a nonreplicate pretest, we first demonstrated that both predator species clearly preferred one option in the olfactometer when choosing between volatiles from clean jack bean leaves and those from spider mite-infested leaves. Each odor source was prepared with two freshly infested bean leaves. Both predators are naturally attracted to volatile compounds released by plants damaged by spider mites. Next, we tested how the two predator species responded to volatiles from leaves infested either only with prey or with prey plus adult females of the heterospecific predator. Two jack bean leaves, each infested with approximately 300 adult females of *T. urticae*, were positioned on water-soaked cotton wool (5 mm deep) at the base of the two jars (4.5 L), after which they were subsequently incubated for 2 days. This setup kept the leaves fresh for several days while the water layer acted as a barrier, preventing mites from dispersing. On one set of leaves, heterospecific predators were introduced (20 or 40 adult females per leaf) and maintained for two days, whereas the other set was left without predators. The odors used in these tests were those emitted by spider mite-infested leaves, which likely included alarm pheromones produced by prey when exposed to predation risk. Additionally, other predator species can trigger the release of these alarm pheromones, which predatory mites may then exploit as cues to avoid plants occupied by different predator species. This experiment was conducted with both predatory mite species and two predator density treatments (20 and 40 adult females per leaf) on bean leaves.

In accordance with the protocol of [39], mated adult females were removed from the culture and starved for 24 h before the experiments. Individual predatory female mites were introduced into the olfactometer, each of which was given a maximum of 5 min to choose one of the arms by reaching the far end of either one of the arms of the olfactory device. Upon choice or expiration of the time limit, the mite was removed and replaced by the next female. Females selecting the right arm were scored as (+), those selecting the left arm as (−), and those not choosing within five minutes were recorded as ‘no choice’. To avoid positional bias, after every set of five females, the air sources were switched between the olfactometer arms, and the metal wire was changed. Each experiment was replicated three to four times with 25–30 predator individuals per replicate/day, depending on availability, and with a new odour source for each trial. After each experiment set, the Y-tube was rinsed with distilled water thoroughly and dried in a hot air oven.

### 2.4. Experience Effects

To assess the influence of prior exposure to heterospecifics on predator behavior, two mixed-species cultures were established separately on bean and cucumber. A single fresh bean or cucumber leaf (15 days post-sowing) was placed on moistened cotton wool inside a plastic Petri dish (13.5 cm diameter), with the petiole inserted into the cotton to maintain leaf turgor. One hundred adult female *T. urticae* were introduced onto each leaf to create prey-infested bean and cucumber colonies. After 24 h, fifty adult females (25 *P. persimilis* and 25 *N. californicus*) were added to each colony. Fresh spider mite-infested leaves of the same plant species were provided daily on top of the initial leaf to ensure a continuous supply of prey and herbivore-induced plant volatiles, while the bottom leaf in contact with the cotton was replaced weekly to prevent wilting.

*P. persimilis* and *N. californicus* females that were reared on one plant species but were exposed to the other plant species (i.e., adult predators from the colony on infested bean leaves were exposed to infested cucumber leaves, or those from infested cucumber leaves were exposed to infested bean leaves), we examined whether experience acquired during rearing influences the responses of predatory mites to volatiles emitted by bean or cucumber plants infested with *T. urticae*, compared with volatiles from plants infested with *T. urticae* in the presence of heterospecific predators. Specifically, we assessed whether predators reared under different conditions differed in their behavioral responses to these odor sources. In a closed-system Y-tube olfactometer assay, predators reared on cucumber leaves infested with *T. urticae* were tested for their reactions to volatile organic compounds emitted from bean leaves infested with approximately 300 adult females of *T. urticae* alone versus those emitted from bean leaves infested with 300 adult females of *T. urticae* and 20 adult females of heterospecific predators, and vice versa. Individual female predators were released at the base of the olfactometer by placing them on a metal wire positioned along the central axis of the tube. Each predatory mite was observed for up to 5 min, and individuals that failed to choose either odor source within this period were scored as ‘no-choice’. To avoid potential positional bias, odor sources were alternated after every five tested predators. Predators from different treatments were tested alternately on the same day using identical odor sources to ensure comparability. Predators were collected from the mixed-species colonies after 1, 2, 3, and 4 weeks of exposure and tested in the olfactometer. Each exposure duration was replicated three times on 4–10 different days, using new sets of odour sources and approximately 20 predators per treatment on each day. Olfactometer experiments were carried out at 25 ± 2 °C.

### 2.5. Statistical Analysis

Predatory adult females that did not make a choice were excluded from the analyses. Olfactometer choices were analyzed using two-sided binomial tests under the null hypothesis of equal distribution between odor sources. The effects of predator treatment, rearing experience, and exposure duration on foraging responses of *P. persimilis* and *N. californicus* were analyzed using a mixed-model via ANOVA (split–split plot) (predicting factors: Weeks of experience + feeding state + predatory mites). Intraguild predation data were analyzed using one-way ANOVA in the absence of shared prey and *t*-tests when shared prey was present. The influence of heterospecific predator density was further evaluated using generalized linear models GLMs [40], with a binomial distribution, with the number of mites choosing *T. urticae*–induced volatiles as the response variable. Additional comparisons were conducted using chi-square and z-tests. All statistical analyses were conducted using SPSS 28.0.

## 3. Results

### 3.1. Intraguild Predation on Heterospecific Prey Without Shared Prey

**Figure 2:**
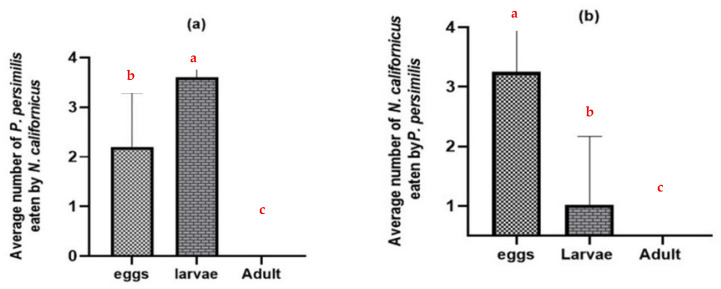
Intraguild predation of predatory mites at different stages in the absence of the shared prey *T. urticae*: (**a**) *N. californicus* predation of *P. persimilis*; (**b**) *P. persimilis* predation of *N. californicus*. The data represent averages and standard deviations from 40 replicates per treatment. (Columns with different letters are significantly different at the 5% level).

Without shared prey, adult females of both predatory mite species consumed the eggs and larvae of the heterospecific predator but did not prey on heterospecific adult females (*p* ≤ 0.0001) (Figure 2). *N. californicus* attacked more *P. persimilis* larvae than eggs (Figure 2a). In contrast, *P. persimilis* caused higher mortality in *N. californicus* eggs than in larval stages (*p* ≤ 0.0001), with all tests conducted at *d.f.* = 2 (Figure 2b).

**Figure 3:**
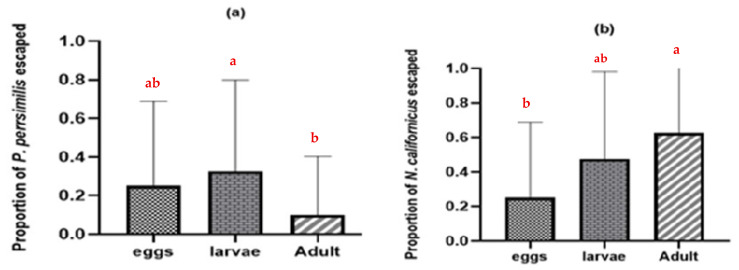
Proportion of adult females that escaped in the absence of shared prey and in the presence of heterospecific eggs, larvae, and adults: (**a**) proportion of escaped *P. persimilis*; (**b**) proportion of escaped *N. californicus*. (Proportions followed by different letters are significantly different at the 5% level).

Additionally, many predatory mite individuals were discovered in the water when they tried to escape leaf arenas containing intraguild prey (Figure 3). The *N. californicus* stages exhibited higher escape rates (*p* ≤ 0.05 *, *d.f.* = 2) than *P. persimilis* stages (*p* ≤ 0.003 **, *d.f.* = 2) when heterospecific adults were present (Figure 3b).

### 3.2. Intraguild Predation on Heterospecifics with Shared Prey

**Figure 4:**
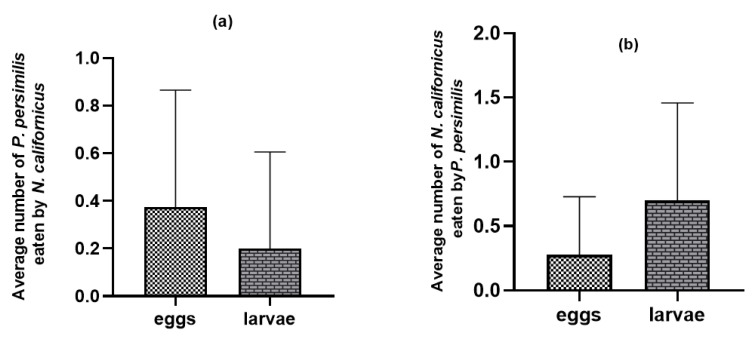
Intraguild predation between predatory mites with *T. urticae* as shared prey: (**a**) *N. californicus* feeding on *P. persimilis*; (**b**) *P. persimilis* feeding on *N. californicus*. The values shown are the means ± standard deviations of 40 replicates per treatment.

On bean leaves where the common prey was present, adult females of both predatory mite species consumed the eggs and larvae of the heterospecific predator (*p* = 0.11 n.s., t = 1.64: *p* = 0.005 **, t = −2.98 for *N. californicus* and *P. persimilis*, respectively). Nonetheless, the frequency of such intraguild predation was relatively low (Figure 4) compared with the substantially higher levels observed in the absence of shared prey (Figure 2).

### 3.3. Behavioral Bioassay

**Figure 5:**
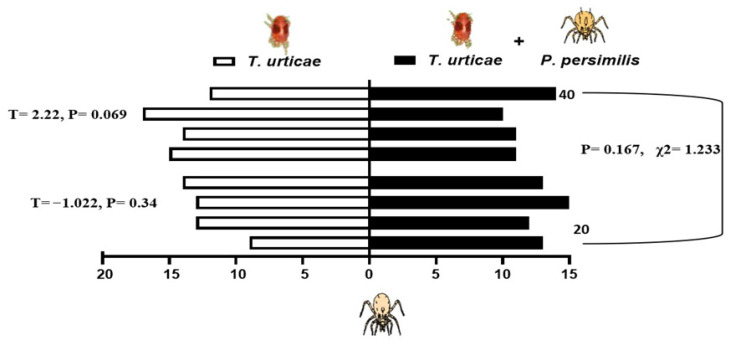
Behavioral response of *Neoseiulus californicus* to plant volatiles: comparison between leaves infested with spider mites alone (left bars) and leaves infested with spider mites plus 20 or 40 *Phytoseiulus persimilis* per leaf (right, black bars). The data are based on four replicates.

**Figure 6:**
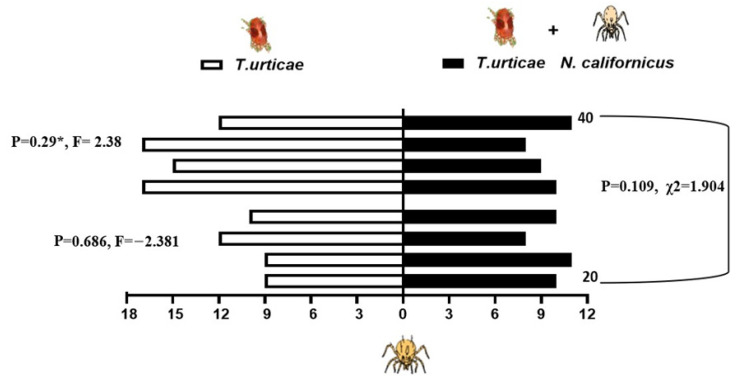
Response of *P. persimilis* to volatiles from plants with spider mites (left side of the bars) and those from plants with spider mites plus *Neoseiulus californicus*, either 20 or 40 per leaf (right side, black bars). Four replicates were performed (*, *p* < 0.05).

When presented with a choice between volatiles emitted from bean leaves infested solely with shared prey, *T. urticae*, and those from leaves harboring both the prey and either 20 or 40 heterospecific predators, the predatory mites showed differential responses depending on the composition of odor sources. *N. californicus* showed no significant preference for either odor source (20 individuals: *p* = 0.34, t = −1.022; 40 individuals: *p* = 0.069, t = 2.216). Similarly, the density of *P. persimilis* did not significantly influence plant choice in the two-sided binomial test (*p* = 0.167, χ^2^ = 1.233, *d.f*. = 1; Figure 5). In contrast, *P. persimilis* displayed a significant preference for bean leaves with prey alone over those with prey plus heterospecifics at the higher density of 40 (*p* = 0.29 *, *F* = 2.38), but this effect was absent when the heterospecific density was 20 (*p* = 0.686, *F* = −2.381; Figure 6). Overall, the heterospecific predator density on leaves did not significantly influence treatment outcomes (χ^2^ = 1.904, *d.f*. = 1, *p* = 0.109).

The combined results were not statistically significant. Among all the *N. californicus* samples tested, 41.80% and 48.33% chose the odor of *T. urticae* only, whereas 45.08% and 38.33% chose the odor of *T. urticae* plus 20 or 40 heterospecific predators, respectively. Similarly, 35.29% of *P. persimilis* chose the odor of *T. urticae* plus 40 heterospecifics, whereas 52.94% chose the odor of *T. urticae* only; 42.86% chose *T. urticae* plus heterospecifics at a density of 20 per leaf. However, no significant difference was found in any of these comparisons.

### 3.4. Experience Effects

#### 3.4.1. Foraging Activity of *P. persimilis* on Cucumber and Bean Leaves Infested with Spider Mites Alone or Accompanied with a Heterospecific Predator

**Figure 7:**
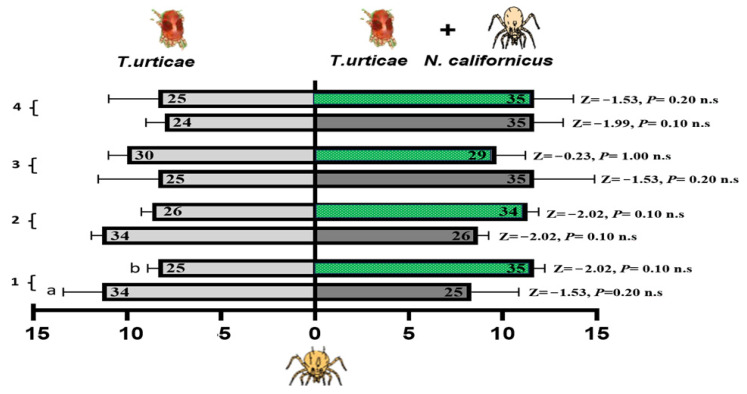
Olfactometer responses of *Phytoseiulus persimilis* when given a choice between volatiles from leaves infested with spider mites (left bars) and those with spider mites plus *N. californicus* (right bars). (a) Cucumber leaves; (b) bean leaves. The bars display the mean number of CU-predators (reared on cucumber with spider mites) and LI-predators (reared on lima bean with spider mites) selecting each odor source. *p* values from Z tests are shown beside each bar for weeks 1–4. The results represent three independent replicates per week, with each bar reflecting the choices of sixty predators; the numbers inside the bars denote responsive individuals (n.s. = non-significant).

Figure 7 shows the foraging responses of *P. persimilis* when exposed to two different odors, one from cucumber and the other from bean leaves infested with either *T. urticae* alone or a combination of *T. urticae* and *N. californicus*. No significant differences were found for either plant type at any of the time points (one, two, three, or four weeks), as indicated by the nonsignificant *p*-values obtained from the Z-tests. The interaction effects of host plant experience, lack of experience, and feeding status on the foraging behavior of *P. persimilis* in response to spider mite prey odor, alone or with *N. californicus*, were evaluated via GLM. Over a range of experience durations (one to four weeks) (Table 1), the results revealed no significant effects of treatment, feeding state, or their interaction in most cases, suggesting that these factors did not consistently influence the foraging behavior of *P. persimilis*.

Notably, during the two-week experimental period, the feeding state significantly affected the response to spider mites (*p* = 0.02 *), with an interaction effect between the treatment and the feeding state (*p* = 0.05 *) at *d.f*. = 1 (Table 1). However, these effects were temporary and diminished with prolonged exposure, as evidenced by nonsignificant results at three and four weeks. These findings highlight that short-term feeding experiences on specific host plants may influence foraging behavior under particular conditions, but their effects do not persist over time.

#### 3.4.2. Foraging Activity of *N. californicus* on Cucumber and Bean Leaves Infested with Spider Mites Alone or Accompanied by Heterospecific Predators

**Figure 8:**
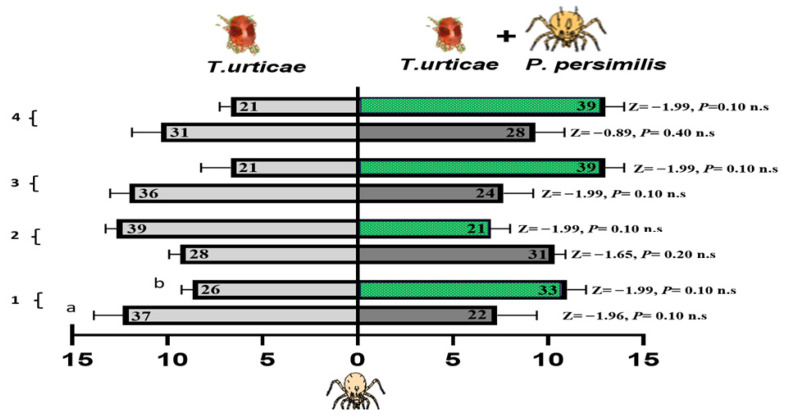
Olfactometer responses of *N. californicus* to volatiles from leaves infested with spider mites (left bars) and those infested with spider mites plus *P. persimilis* (right bars). (a) Cucumber leaves; (b) bean leaves. The bars display the mean number of CU-predators (reared on cucumber with spider mites) and LI-predators (reared on lima bean with spider mites) selecting each odor source. *p* values from Z tests are shown beside each bar for weeks 1–4. The results represent three independent replicates per week, with each bar reflecting the choices of sixty predators; the numbers inside the bars denote responsive individuals (n.s. = non-significant).

*N. californicus* exhibited no preference for volatiles emitted from spider mite-infested cucumber or bean leaves or from leaves infested with both spider mites and *P. persimilis* (Figure 8). Across all the treatments and time points, no significant differences were detected, as all the *p*-values for the Z tests were nonsignificant. These results indicate that *N. californicus* does not differ in olfactory cues related to the presence of heterospecific predators, regardless of the host plant type or duration of exposure. The results in Table 2 indicated that the foraging response of *N. californicus* is significantly influenced by the interaction between host plant experience (or lack thereof) and feeding conditions. Statistical analysis via generalized linear models (GLMs) revealed consistent and significant interaction effects (A × B) throughout the experimental period for both spider mites alone and for spider mites combined with heterospecific predators. For instance, during the first week, the interaction term for spider mites was significant (*p* = 0.006), highlighting the strong combined effect of treatment and feeding state. A similar significant interaction was observed for spider mites and heterospecific mites, with a *p*-value of 0.006.

As the experiment progressed, the treatment effects became more pronounced, with significance levels increasing over time. Following the exposure of cucumber or bean leaves to *T. urticae* and *P. persimilis* over one to four weeks, *N. californicus* displayed a clear preference for volatiles from both types of infested leaves at four weeks of experience (*p* = 0.006, for spider mites; *p* = 0.002, for spider mites plus heterospecific mites). This finding suggests a significant influence of host plant experience on predator behavior. Although the feeding state had significant effects earlier in the experiment, particularly at two weeks (*p* = 0.003, F = 18.75 for spider mites), its influence diminished by four weeks, with nonsignificant results (*p* = 0.19, F = 2.00) all at *d.f.* = 1.

These statistical trends underscore the dynamic nature of *N. californicus* foraging behavior; however, while the feeding state initially plays a critical role, the host plant experience becomes increasingly important. The persistent significance of the interaction terms (*p* < 0.05 across weeks) demonstrates that the combined effects of these variables are more influential than their individual contributions. These results highlight the importance of considering the feeding state and plant experience when designing effective biological control strategies.

#### 3.4.3. Interaction Between Feeding State, Weeks Experiences, and Predator Mites on the Foraging Behavior of Predatory Mites

The data in Table 3 summarizes how feeding state, experience duration (weeks), and predator species interact to influence behavior and response time of predatory mites when exposed to volatiles emitted by leaves infested with spider mites alone or spider mites with heterospecific predators.

Overall, the predator species exerted the strongest and most consistent effect across all tested conditions. Significant differences were detected between *P. persimilis* and *N. californicus* in their responses to the two odor sources and in the time taken to make a choice, indicating species-specific foraging strategies. In some cases, the duration of experience alone influenced predator responses, particularly toward leaves infested with spider mites alone, suggesting that prior exposure can gradually modify foraging behavior. However, experience duration by itself did not consistently affect all response variables.

In contrast, the feeding state (fed vs. starved) had no significant main effect on predator choice or response time. Nevertheless, the feeding state became important when combined with other factors. Significant interactions between feeding state, experience, and predator species indicate that hunger effects depend on both the predator identity and its previous experience. Notably, the three-way interaction (predator species × feeding state × weeks of experience) was highly significant for all response variables. This demonstrates that predator behavior is shaped by a complex combination of intrinsic traits and external conditions rather than by single factors acting independently.

Taken together, these findings underscore the behavioral flexibility of predatory mites and demonstrate that their foraging decisions are shaped by species identity, prior experience, and physiological condition, particularly in the presence of heterospecific competitors. They further emphasize the importance of accounting for feeding state and host-plant experience when developing and optimizing biological control strategies.

## 4. Discussion

The use of multiple natural enemies for the control of different pest species has transformed crop systems from relatively simple predator–pest–plant interactions into complex food-web structures. When multiple predatory mite species are attracted to plants infested with the same prey, their interactions may involve food competition, intraguild predation, or behavioral interference, all of which can alter their foraging behavior and ultimately affect biological control outcomes. Our results demonstrated that when no alternative prey was available, adult female predatory mites attacked and consumed heterospecific eggs and larvae but avoided heterospecific adults, with intraguild predation risk remaining relatively low. This stage-specific pattern is consistent with earlier findings by Çakmak et al. (2006) [2], who reported that adult females of both species preferentially targeted heterospecific eggs and larval stages and avoided adults, accompanied by frequent escape behavior of intraguild prey. In line with these observations, we found that juvenile *N. californicus* exhibited higher escape rates than juvenile *P. persimilis*, suggesting species-specific differences in antipredator responses. Importantly, when shared prey was available, intraguild predation was markedly reduced, indicating that prey availability buffers antagonistic interactions between these predators. Similar reductions in intraguild predation in the presence of shared prey have been reported by Walzer and Schausberger (1999a,b) [41,42], who demonstrated contrasting predation strategies between the generalist *N. californicus*, which feeds indiscriminately on heterospecific larvae, and the specialist *P. persimilis*, which feeds discriminately. *P. persimilis* prioritizes *T. urticae* as prey, whereas *N. californicus* exhibits a flexible predation pattern, targeting both spider mites and other predatory mites. This dietary plasticity may reduce direct competition and facilitate coexistence. According to our results, the antipredator behavior of intraguild prey toward intraguild predators is often triggered by volatile chemical cues [8], which promotes escape behaviors aimed at avoiding predation. Similarly, Janssen et al. (2007) [43] demonstrated how habitat structure modulates intraguild predation dynamics. Although *N. californicus* can prey on *P. persimilis* juveniles, actual intraguild predation is minimal when shared prey are available, reflecting the preference of *N. californicus* for spider mites [2]. Holt and Polis (1997) [44] provided a theoretical basis for understanding these intricate interspecific interactions, emphasizing both the direct and indirect influences of intraguild predation on prey dynamics. Empirical studies have shown that intraguild predation can compromise the performance of specialist natural enemies in biological control programs [45,46], although the degree of impact often depends on the surrounding ecological context [47]. In summary, the behavior of intraguild prey in response to intraguild predators, including avoidance and escape strategies, is a critical aspect of predator–prey interactions. To fully grasp these complex dynamics, it is essential to consider both habitat structure and the direct and indirect consequences of intraguild predation.

Our results demonstrate that both *N. californicus* and *P. persimilis* are strongly attracted to volatiles emitted from jack bean or cucumber leaves infested by spider mites, confirming the central role of herbivore-induced plant cues in predator foraging. Despite this strong attraction, neither species consistently avoided odors from plants simultaneously harboring spider mites and heterospecific predators, even at higher predator densities. This pattern indicates that the presence of a competing predator does not necessarily reduce the attractiveness of prey-infested plants. Similar findings were reported by Çakmak et al. (2006) and Pallini et al. (1999) [2,48], who showed that predator presence does not always trigger avoidance responses and that recognition of predators may depend on species-specific traits and local biological control histories. Likewise, Fonseca et al. (2010) [23], found that *N. californicus* and *P. macropilis* were equally attracted to spider mite-infested plants regardless of competitor presence, supporting the view that these predators do not avoid potential competitors during foraging. Consistent with our results, greenhouse release experiments also demonstrated that *P. persimilis* does not discriminate between plants infested solely with spider mites and those additionally containing *N. californicus* [49]. In contrast, other studies have documented avoidance behavior mediated by plant-derived volatiles, whereby predatory mites actively avoid patches occupied by conspecifics or heterospecific competitors, for example, Gnanvossou et al. (2003) and Janssen et al. (1997) [5,50] demonstrated that predator presence can reduce patch attractiveness, leading to spatial separation among competing species. Similarly, Maleknia et al. (2013), Magalhães et al. (2005), and Bayoumy and Ramadan (2018) [6,8,51] showed that avoidance behavior indicates that volatile cues help drive these responses, highlighting the complex nature of these ecological interactions. Furthermore, Gnanvossou et al. (2003) and Onzo et al. (2003) [5,52] reported that three predatory mite species—*T. aripo*, *Typhlodromalus manihoti*, and *Euseius fustis*—avoid patches with either conspecifics or heterospecifics, with *T. manihoti* favoring patches with *T. aripo* over conspecifics. These behaviors indicate that predatory mites use volatiles to assess prey patch profitability, affecting their interactions and population dynamics in natural settings. The plant-emitted volatiles induced by herbivore attack not only attract predators but also influence interspecific predator interactions [53]. A thorough understanding of bottom-up factors—especially host–plant defenses that impact prey quality—is essential for optimizing biological control [54]. Predator responses to plant-derived chemical cues are further influenced by prior experience and physiological state [55,56,57], with predatory mites integrating kairomones from prey–plant interactions and synomones released by host plants [58].

Contrary to our initial expectation, *P. persimilis* did not consistently avoid patches containing *N. californicus* based on prior experience, as previous exposure exerted only a limited and context-specific influence on foraging decisions. Statistical analyses revealed variable effects of treatment and feeding state, with significant interactions occurring only under particular experimental conditions, indicating that experience-dependent responses are not uniform. These results are consistent with the view proposed by Vet and Groenewold (1990) [59] that disentangling adult behavioral plasticity from juvenile experience remains inherently difficult. Taken together, our findings reinforce the idea that plant-mediated and prey-related chemical cues play a dominant role in shaping predatory mite foraging behavior, while postembryonic experiences have a stronger influence than parental or embryonic factors, as reported by Zhang and Sanderson (1992) and Sznajder et al. (2011) [60,61]. Moreover, the observed patterns in predator responses qualitatively match the variable-response model described by Vet et al. (1990) [62], which predicts flexible and experience-dependent decision-making in foraging natural enemies. These insights have important implications for biological control of spider mites, particularly because *P. persimilis* is routinely mass-reared on bean plants and released across a wide range of crops, where prior rearing conditions may only weakly shape post-release foraging behavior. However, during transportation in bran-filled containers, these predators may lack access to plant cues. They could experience hunger, raising questions about how these conditions might impact their effectiveness upon release. Ref. [57] indicated that while herbivore-induced plant volatiles (HIPVs) can attract the natural enemies of herbivores, the ability of these predators to discriminate between different HIPV blends is influenced by their prior experiences and feeding states. Although short-term exposure to specific herbivore cues may enhance foraging responses, this effect diminishes over time, suggesting that predators may benefit from exploiting plants already infested by unsuitable herbivores, leading to potential population stability in predator–prey dynamics. Ref. [63] investigated the learning capabilities of the predatory mite *P. persimilis*. They concluded that such abilities enable it to forage successfully in habitats where prey occurs on plant species different from those used during predator development and even in environments containing non-prey caterpillars.

Earlier investigations [64,65], along with work by Krips et al. (1999) and Zhang et al. (2022) [66,67], have shown that prior exposure shapes how *P. persimilis* reacts to plant-derived volatile cues. In particular, exposure history can alter how predatory mites react to odors from spider mite–infested leaves, especially when those volatiles are encountered alongside heterospecific predators. For example, if *N. californicus* has been previously exposed to a mixed predator environment, its olfactory sensitivity or foraging strategies may be altered when it reencounters similar conditions [68]. This adaptability is crucial for optimizing their role in integrated pest management strategies. Çakmak et al. (2006) [2] reported that when *P. persimilis* and *N. californicus* were jointly reared for one to four weeks on bean leaves infested with spider mites and heterospecific predators, neither mite species showed a distinct preference for volatiles from leaves infested only with spider mites versus those with heterospecific predators. Drukker et al. (2000a) [55] observed that food-deprived *P. persimilis* exposed to volatiles induced by *T. urticae* later avoided these odor cues, suggesting that mites can acquire avoidance behavior through non-rewarding experiences. Differences in rearing protocols may explain the contrasting outcomes across studies, as Drukker et al. (2000a) [55] kept predators without exposure to volatiles, whereas Çakmak et al. (2006) [2] maintained them in volatile-rich environments. Prior exposure to one set of volatiles can suppress or interfere with learning responses to other odor cues, a pattern also documented in honeybees (*Apis mellifera*) during olfactory conditioning [69]. It is still unclear whether predatory mites experience similar interference. Ecologically, the weaker influence of non-rewarding experiences than rewarding experiences is noteworthy. While a rewarding cue reliably indicates prey availability, a nonrewarding cue may not consistently reflect prey absence, particularly across broader spatial contexts [70].

## 5. Conclusions

This study shows that intraguild predation between the two predatory mites, *P. persimilis* and *N. californicus*, which primarily prey on eggs and larvae, is substantially reduced when shared prey are present. Both predatory mite species respond positively to herbivore-induced plant volatiles and do not avoid patches containing heterospecific competitors. Although prior experience and feeding state can influence short-term foraging behavior, these effects appear to be transient. Overall, the combined use of specialist and selective predatory mites is compatible with integrated biological control strategies, as the risk of intraguild predation remains low when prey densities are sufficient, supporting their combined application in integrated pest management (IPM) programs.

## Figures and Tables

**Table 1:** Influence of host plant experience and feeding state on the foraging behavior of *P. pesimilis* toward leaves infested with spider mites alone or spider mites plus heterospecific predators. The data were analyzed via generalized linear models (GLMs). Significant values are shown in bold. (*, *p* < 0.05; n.s. = not significant).

		Spider Mites			Spider Mites + Heterospecific		
Experience (A)	Source of variation	Mean square	*F*. value	*p*. value	Mean square	*F*. value	*p*. value
One week	Treatment (A)	0.33 a	0.17	0.08 n.s.	0.08 a	0.03	0.56 n.s.
	Feeding state (B)	8.33 a	4.17	0.69 n.s.	10.08 a	4.03	0.08 n.s.
	A*B	5.33	2.67	0.14 n.s.	6.75	2.7	0.14 n.s.
Two weeks	Treatment (A)	0.33 b	0.33	0.58 n.s.	0.08 b	0.08	0.78 n.s.
	Feeding state (B)	8.33 a	8.33	0.02 *	6.75 a	6.75	0.03 *
	A*B	5.33	5.33	0.05 *	4.08	4.08	0.08 n.s.
Three weeks	Treatment (A)	3.00 a	0.82	0.39 n.s.	2.08 a	0.52	0.49 n.s.
	Feeding state (B)	1.33 a	0.36	0.56 n.s.	2.08 a	0.52	0.49 n.s.
	A*B	3.00	0.82	0.39 n.s.	4.08	1.02	0.34 n.s.
Four weeks	Treatment (A)	14.08 a	4.97	0.06 n.s.	8.33 a	3.70	0.09 n.s.
	Feeding state (B)	0.08 a	0.03	0.86 n.s.	0.0 a	0.00	1.00 n.s.
	A*B	0.03	0.03	0.87 n.s.	0.33	0.15	0.71 n.s.

Notes: A. Treatment refers to the state of the predator, i.e., no experience or experience. B. The feeding state refers to “reared on cucumber or bean” (means with different lower-case letters indicate significant differences with *p* < 0.05).

**Table 2:** Impact of host plant experience and feeding state on *N. californicus* foraging responses on the side with spider mites or on the side with spider mites plus heterospecific mites. Generalized linear models (GLMs) were used to analyze the data. *, *p* < 0.05; **, *p* < 0.01, ***, *p* < 0.001; n.s. = not significant.

		Spider Mites			Spider Mites + Heterospecific		
Experience (A)	Source of variation	Mean square	*F*. value	*p*. value	Mean square	*F*. value	*p*. value
One week	Treatment (A)	1.33 b	0.89	0.37 n.s.	2.08 a	1.19	0.31 n.s.
	Feeding state (B)	3.00 a	2.00	0.19 n.s.	2.08 a	1.19	0.31 n.s.
	A*B	21.33 a	14.22	0.006 **	24.08	13.76	0.006 **
Two weeks	Treatment (A)	0.08 b	0.08	0.78 n.s.	0.33 a	0.44 a	0.52 n.s.
	Feeding state (B)	18.75 a	18.75	0.003 **	21.33 b	28.44	0.0007 ***
	A*B	2.08	2.08	0. 19 n.s.	1.33	1.78	0. 22 n.s.
Three weeks	Treatment (A)	10.08 a	7.12	0.029 *	12.00 b	12.00	0.02 *
	Feeding state (B)	10.08 b	7.12	0.029 *	8.33 a	8.33	0.009 **
	A*B	36.75	25.94	0.0009 ***	40.33	40.33	0.0002 ***
Four weeks	Treatment (A)	21.33 a	14.22 a	0.006 **	24.08 b	19.27	0.002 **
	Feeding state (B)	3.00 b	2.00 b	0.19 n.s.	2.08 a	1.67	0.233 n.s.
	A*B	21.33	14.22	0.006 **	24.08	19.27	0.002 **

A. Treatment refers to the state of the predator, i.e., non-experiences or experiences. B. The feeding state refers to “reared on cucumber or bean”. (means with different lower-case letters indicate significant differences with *p* < 0.05).

**Table 3:** The interaction between feeding state, weeks experiences, and predator mites on the foraging behavior of predatory mites toward leaves infested with spider mites alone or spider mites plus heterospecific predators and time response.

Factors		Predators Response			
		*Tertranychus urtica*	*T. u* + Hetterospicific	(*T. u*) Time	(*T. u* + Hetterospicifi) Time
Weeks	*d.f*.	3	3	3	3
	F	5.59	4.35	0.67	4.78
	*P*	0.04 *	0.55 n.s.	0.06 n.s.	0.05 *
Feeding state	*d.f*.	1	1	1	1
	F	3.72	4.72	4.72	1.99
	*P*	0.09 n.s.	0.06 n.s.	0.062 n.s.	0.196 n.s.
Feeding state × Weeks	*d.f*.	3	3	3	3
	F	0.56	0.42	1.49	8.08
	*P*	0.66 n.s.	0.75 n.s.	0.29 n.s.	0.008 **
Predatory mites	*d.f*.	1	1	1	1
	F	25.99	28.09	8.26	38.58
	*P*	0.0002 ***	0.0001 ***	0.012 *	0.0001 ***
Predatory mites ×Weeks	*d.f*.	3	3	3	3
	F	6.46	6.68	2.12	7.77
	*P*	0.006 **	0.005 *	0.14 n.s.	0.0001 ***
Predatory mites × Feeding state	*d.f*.	1	1	1	1
	F	7.07	8.41	6.27	6.02
	*P*	0.02 *	0.0116 *	0.03 *	0.028 *
Predatory mites × Feeding state × Weeks	*d.f*.	3	3	3	3
	F	18.85	16.81	13.90	10.17
	*P*	0.0001 ***	0.0001 ***	0.0002 ***	0.0008 ***
R2		92%	91.38%	89.02%	90.25%
Coefficient Variation		12.18	12.45	18.29	15.37

The data were analyzed via Anova (Split-Split Plot). (predicting factors: Weeks of experience + feeding state + predatory mites) (*, *p* < 0.05; **, *p* < 0.01; ***, *p* < 0.001; n.s. = not significant), (*T. u* = *Tetranychus urticae*).

## Data Availability

The data presented in this study are available on request from the corresponding author. The data are not publicly available due to privacy restrictions.
